# Sentimental Analysis of Twitter Users from Turkish Content with Natural Language Processing

**DOI:** 10.1155/2022/2455160

**Published:** 2022-04-13

**Authors:** Cagla Balli, Mehmet Serdar Guzel, Erkan Bostanci, Alok Mishra

**Affiliations:** ^1^Department of Computer Engineering, Ankara University, Ankara 06830, Turkey; ^2^Faculty of Logistics, Molde University College-Specialized University in Logistics, Molde 6402, Norway; ^3^Software Engineering Department, Atilim University, Ankara 06830, Turkey

## Abstract

Artificial Intelligence has guided technological progress in recent years; it has shown significant development with increased academic studies on Machine Learning and the high demand for this field in the sector. In addition to the advancement of technology day by day, the pandemic, which has become a part of our lives since early 2020, has led to social media occupying a larger place in the lives of individuals. Therefore, social media posts have become an excellent data source for the field of sentiment analysis. The main contribution of this study is based on the Natural Language Processing method, which is one of the machine learning topics in the literature. Sentiment analysis classification is a solid example for machine learning tasks that belongs to human-machine interaction. It is essential to make the computer understand people emotional situation with classifiers. There are a limited number of Turkish language studies in the literature. Turkish language has different types of linguistic features from English. Since Turkish is an agglutinative language, it is challenging to make sentiment analysis with that language. This paper aims to perform sentiment analysis of several machine learning algorithms on Turkish language datasets that are collected from Twitter. In this research, besides using public dataset that belongs to Beyaz (2021) to get more general results, another dataset is created to understand the impact of the pandemic on people and to learn about public opinions. Therefore, a custom dataset, namely, SentimentSet (Balli 2021), was created, consisting of Turkish tweets that were filtered with words such as pandemic and corona by manually marking as positive, negative, or neutral. Besides, SentimentSet could be used in future researches as benchmark dataset. Results show classification accuracy of not only up to ∼87% with test data from datasets of both datasets and trained models, but also up to ∼84% with small “Sample Test Data” generated by the same methods as SentimentSet dataset. These research results contributed to indicating Turkish language specific sentiment analysis that is dependent on language specifications.

## 1. Introduction

Artificial intelligence is simply defined as machines trying to imitate human intelligence and behavior. Machine learning is computer models that learn big data and make inferences from them [1]. These models consist of a series of steps that are based on statistical algorithms, process big data, and make predictions with the mathematical results it draws from them [2]. These models can be iterated and used for different data, improved with different algorithms, or retrained with different hyperparameters to get better results. It can be reused without retraining with different test data using the trained model. The goal is always to make a better guess and get better results.

Natural language processing (NLP) is a subfield of linguistics, computer science, and artificial intelligence concerned with interactions between computer and human language, specifically how to program computers to process and analyze natural language data. Its aim is to obtain a computer that can “understand” the content, along with the idiosyncratic aspects of the natural language used in writing the texts. Afterward, it is possible to classify and edit the information in the text content and extract the information correctly [3].

Sentiment analysis [4] is a field that computer science and linguistics use together that aims to determine the sentiment contained in written data. In general, sentiment analysis algorithms are used to classify datasets by dividing them into different categories or classes [5]. For the studies in this field, working with machine learning algorithms, for these meanings extracted from the data, such as writing techniques, language tools, linguistic developments for different languages, interpretation of different meanings of the word in linguistics, and the change of the emotion expressed when words come together, has produced very successful results. Besides, there are text classification studies [6, 7] in the literature that also worked on text and documents; however, they are aimed to find useful information for business intelligence instead of emotions [8].

Today, one of the most common and most diverse data sources used for sentiment analysis is social media. Social media offer to be important data source [9] in current big data studies, where the data is constantly renewed instantly, information on current issues spreads rapidly between societies and people, and it is full of different perspectives on every subject such as politics, science, and history. Twitter is one of the best source websites and also a popular microblogging forum [10] for providing written data on current topics or big topics in social media.

Sentiment analysis is a very common topic in the literature and worldwide; many studies have been done on this subject. However, sentiment analysis has strong dependency on the linguistic features since it is based on the language of a text, and modelling is established by a text from the same language [11]. There are many successful studies done for texts in English language. As an alternative to the language dependency, there is a study that belongs to Denecke [12]; to translate a language to English before doing sentiment analysis regardless of language is used for text. However, in general, sentiment analysis studies are done individually per language such as [13, 14].

Machine learning algorithms [11, 15–31] were commonly used for Turkish sentiment analysis problems in previous studies. Bozyigit et al. [19] present a study that used LSTM and different CNN networks for Turkish sentiment analysis over Turkish user comments. SVM is used by Kaya et al. [15] in 2012 on a study of sentiment analysis for Turkish political news. In the study of Coban et al. [17] in 2015, Twitter data were used for sentiment analysis according to emojis using various machine learning algorithms such as SVM, Naive Bayes, Multinomial Naive Bayes, and KNN.

Although, in recent years, the number of Turkish studies has increased, there is still a need for more in the literature to provide variety. In this study, it is aimed to indicate accuracy of various machine learning algorithms on Turkish sentiment analysis with using different datasets and preprocessing steps over Twitter data. Unlike other similar studies, the study is conducted with not only a public dataset (Beyaz [32]), but also a custom dataset (SentimentSet [33]) that is created by using social media with custom topic, which is “pandemic.” This multicombination study could be useful in increasing the accuracy or validation of the research to compare results of the machine learning algorithms with Turkish language sentiment analysis. Moreover, it will contribute with other Turkish studies that already exist and give insights for the next researchers about compatibilities of machine learning algorithms with the sentiment analysis in Turkish language.

In this study, which is conducted in Turkish, two datasets that consist of twitter data are labeled as positive, negative, and neutral, while the training models with these datasets neutral tweets are ignored. The marked (labeled) data was preprocessed with various libraries according to the language characteristics specific to Turkish. Afterwards, datasets are used to train Machine Learning models with various algorithms, and predictions were made on the test data with these models. The results are compared between different combinations of the datasets, algorithms, and different preprocessing libraries.

The remainder of this paper consists of the following parts. In Section 2, the literature review is briefly represented.  Section 3 introduces the methodology that is used for the sentiment analysis models and datasets in detail.  Section 4 explains the experimental studies, as well as the results and metrics of the different machine learning models per datasets.  Section 5 compares the results of similar studies from the literature and gives authors insights about the study.  Section 6 shows the conclusion and prospects.

## 2. Literature Review

Although the beginning of Artificial Intelligence dates back to very old times, the beginning of Natural Language Processing is a subject that dates back to ancient times and is now a subbranch of Artificial Intelligence in the field of Machine Learning. Natural Language Processing was first published by Alan Turing [34] in 1950, a seminal paper on Artificial Intelligence known as the Turing Test. Turing had set the machine's task and intelligence criterion to be the automatic interpretation and generation of natural language. But it was not yet studied under Natural Language Processing at that time. Afterward, John Searle's [35] paper titled Minds, Brains, and Programs, in which he put forward the Chinese Room Experiment, published in 1980, studied the imitation of NLP tasks by the computer when a set of rules, such as a Chinese learning guide, was given to the computer.

Due to the continuous increase in computational power and the emergence of Machine Learning algorithms, there has been a great development in the field of Natural Language Processing. Systems created with the use of these algorithms since the late 1980s were included under the heading Statistical NLP. In automatic speech recognition using statistical methods, they made a great impact with the article “A Maximum Likelihood Approach to Continuous Speech Recognition,” published by Bahl et al. [36] in 1983. According to the article, a maximum likelihood decoding formulation was created for the speech recognition task. In the study, a number of statistical models are explained for use in the speech recognition task. Another statistical study of the 1990s, A Statistical Approach to Machine Translation published by Brown et al. [37], is one of the important researches in this field. In the article, a statistical translation approach from French to English is presented using Bayes' theorem.

Machine Learning algorithms, fast computers, and artificial intelligence networks that are developing day by day can be used in this field. Models created using Deep Neural Networks together with the developing Machine Learning algorithms have begun to become widespread and used in the field of NLP. The Natural Language Processor with Neural Networks (NALPRONN) model, developed by Martinez [38] in 1995 using artificial neural networks in the field of NLP, is one of the milestones in the field of Neural NLP. The NALPRONN system is a multimodal multilingual computer interface. It is a system of artificial neuron networks. This system has processing modules that include the backpropagation network such as I/O displays, input subsystem, output subsystem, dictionary subsystem, and monitor subsystem. There are also memory modules with feature mapping networks. According to the system, tasks are performed by modules. These modules are trained independently of each other with the same data. With NALPRONN, a generalized NLP system has been introduced using artificial neural networks.

In this study, NLP tasks are performed using supervised learning. For this reason, the study focuses on examining the studies conducted with supervised learning in the literature. One of the studies dealing with the sentiment analysis task of NLP using supervised learning is the article “Sentiment Analyzer: extracting sentiments about a given topic using natural language processing techniques” published by Yi et al. [39] in 2003. In the article, two subjects, namely, digital camera and music review, were defined while creating data. Documents collected under these two data groups are randomly selected documents from web pages collected from web scans. These documents were mixed and randomly placed under the subject headings. Two different labels were made for each subject. Subject-related documents are marked as (D+), while off-topic documents are marked as (D−). Various feature selection algorithms such as the Mixture model and Likelihood test were used with this data system. One of the studies carried out using supervised learning for text classification within the scope of the study is the comparison of SVM with kNN Decision Tree and Naive Bayesian methods by Liu et al. [40] in 2010. In the study, an SVM-based classification model is proposed. As a result of these experiments with other given algorithms, it was revealed that the F1 value of the SVM classifier exceeds 86.26%. Another sentiment analysis study using supervised learning is the article titled “A Sentiment Analysis Model For Hotel Reviews Based on Supervised Learning,” published by Shi and Li [41] in 2011. By considering the hotel reviews of the users, it was tried to classify the emotions with a machine learning approach. SVM, one of the supervised learning algorithms, was used in the study.

There are several machine learning algorithms that can be applied to sentiment analysis. Besides, neural networks are also commonly used lately under the sentiment analysis topic. Yao and Guan [42], in 2020, proposed an advanced NLP method. This method was based on the LSTM structure. In the study, compared to Basic and other LSTM, the improved method has better F1 score results in the Wall Street Journal dataset; it is concluded that the revealed method is more suitable for NLP when there are limited computing resources and a large amount of data.

Data collecting is a very important part of the sentiment analysis. Feldman [43] said that Twitter and Facebook are focal points of many sentiment analysis applications in 2013. Since then, Twitter became even the most important data source. Twitter provides user data as anonymous, which is eligible for the sentiment analysis. There are many sentiment analysis studies [44–46] that used Twitter in the literature with several algorithms. In the study on Twitter sentiment analysis conducted by Tam et al. [46] in 2021, an accuracy rate of 91.13% was obtained in the classification performed by using CNN and bidirectional LSTM (Bi-LSTM) models together on tweets in English. However, topic selection is the first step of the collecting data for sentiment analysis. COVID-19 has been a hot topic all over the world since its start. Hence, it took its place in the literature as well. There are COVID-19 related sentiment analysis studies [47–51] that are conducted with Twitter API.

Although there are many articles on sentiment analysis in the literature with COVID-19 topic or in general, there are not as much as studies in Turkish. However, the number of studies in the Turkish language has increased in recent years. In the study of Kaya et al. [15] in 2012 on Sentiment analysis on Turkish political news, political news from different sites of Turkish news were collected. Four algorithms within the scope of supervised learning were compared for emotion classification. These are Naïve Bayes, Maximum Entropy, SVM, and the character-based N-Gram Language Model. From the empirical findings, it was observed that the Maximum Entropy and N-Gram Language Model outperformed SVM and Naive Bayes. By using different features, it has been demonstrated that all approaches reach 65% to 77% accuracy rates.

In the study conducted by Akba [16] in 2014, an F1 score of up to 83.9% was obtained over the models trained with film evaluations. Results were measured with the Information Gain and Chi-Square metrics. Zemberek was used as a preprocess while creating the data in the study, and SVM was used for the classification of the data. In the study of Coban et al. [17] in 2015, the accuracy of up to 66% was obtained by tagging tweets received on Twitter according to emojis and classifying them with various machine learning algorithms using two different feature extraction methods, Bag of words and N-gram model. In another study conducted by Karamollaoglu et al. [18] in 2018, sentiment analysis processes were applied to user comments collected from various websites using the Lexicon-Based method. The classification and sentiment analysis process were carried out with an average success rate of 80%.

Another study conducted in Turkish is the Turkish cyberbullying detection published by Bozyigit et al. [19] in 2019. Artificial neural networks were used in the study. Existing libraries were not used for Turkish Natural Language Processing. In the study, a list named “Harmful Terms” was created, and the wrong spellings between the term and the input were tried to be corrected with the Levenshtein algorithm. For the text mining section, the TF-IDF method that is mentioned in this study was used. Two hidden layers are predicted for the Neural network. As a result of the study, an F1 score of 91% was obtained.

In the study carried out by Pervan [20] in 2019 with LSTM and different CNN networks, using the word2vec model on Turkish user comments collected from the websites, an accuracy value of up to 94% was obtained in LSTM.

One of the NLP studies on Turkish is a sentiment analysis study conducted with Machine Learning algorithms, published by Rumelli et al. [21] in 2019. During the study, open-source libraries such as Zemberek [22] prepared on Turkish Natural Language Processing were used. The data received from an e-commerce website are marked as negative, neutral, and positive according to the scores given by the users and trained with Machine Learning algorithms such as Naive Bayesian, Random Forest, and SVM. As a result of the study, a score of 73.8% was obtained.

A Corpus of Turkish Offensive Language on Social Media, published by Coltekin [23] in 2020, is a study to detect Turkish offensive language in social media. In the study, a dataset consisting of 19% of messages labeled as offensive language was used. A 77.3% F1 score was obtained with the linear SVM. Another Turkish study conducted in recent years is the sentiment analysis of people on global warming and climate change, conducted by Kirelli and Arslankaya [11] in 2020.

Although there are a limited number of Turkish language sentiment analysis studies, it increased in recent years [24, 25]. Aydogan and Kocaman [26] offered a new dataset since there are limited Turkish datasets to work on. Lately, some COVID-19 related studies [28–31] can be found in the literature.

The accuracy values of some of the sentiment analysis studies on Turkish in the literature are shown in Table 1.

According the literature review, our main contribution is providing a Turkish sentiment analysis, which is limited in number in the literature over COVID-19 topic. Besides, in our study, a pandemic topic based dataset was created as benchmark dataset to be used also by not only us, but also future researchers. Moreover, another public dataset that belongs to Beyaz [32] was also used for the sentiment analysis. There is no other study published in the corresponding literature using this public dataset. Finally, several machine learning algorithms such as SVM, Logistic Regression, and LSTM with applying different preprocessing techniques were used together, and the results were compared.

## 3. Methodology

**Figure 1:**
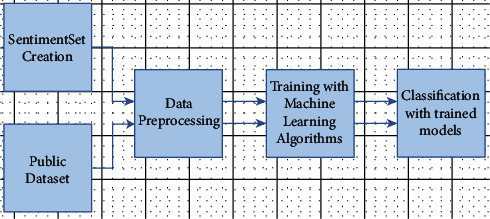
Model creation architecture general schematic.

Two different datasets were used for working with text data as a natural language in order to conduct sentiment analysis. The Public dataset has big amount of data that is already tagged and ready to use, which can provide more accurate results with variety of the data. This dataset also has no data that is relevant to the pandemic. It is compared with inhomogeneous SentimentSet dataset, which is created in the scope of this study and has mostly negative data. These datasets consist of pretagged positive and negative tweets that are gathered from Twitter. The datasets are trained with various Machine Learning algorithms. The emotional states of social media users were tried to be classified as positive or negative using that trained models. The architecture of model generation that includes dataset preparation, training, and classification process is shown in Figure 1. After creating these models, the success rates of the algorithms used and the results obtained in this research were compared, and the accuracy rates were revealed.

**Figure 2:**
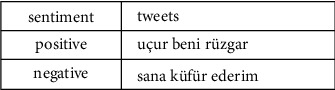
Sample data that belongs to the public dataset.

The public dataset used within the scope of the study is an open dataset developed by Beyaz [32] in 2020 for a project on the detection of bullying in social media and was put into use as a public dataset that underwent various preprocesses. This dataset, which contains approximately 11,000 data, consists of Turkish tweets marked as positive and negative. It is seen that there are already some preprocesses in the dataset used, but in this study, the data was preprocessed again while creating the models. An example of the public dataset is shown in Figure 2.

In addition to the public dataset used for training the models in this study, the second dataset used is the SentimentSet developed within the scope of the study. The dataset consists of approximately 2600 Turkish tweets. Each tweet is marked as positive, negative, or neutral. Two methods were used while collecting the tweets that make up the dataset. The first of these methods is the use of the stream method of the Twitter API, and the second is the use of the open-source snscrape [52] library. Tweets are randomly selected without following any order. The first method used for collecting the tweets is the Stream method, which belongs to the Tweepy library and provides data in accordance with the given parameters. The dataset was created from real-time tweets selected by searching the words “aşı”, “aşılanmak”, “aşı olmak”, “vaccine”, “vaccinated”, “vaccination”, “stayhome”, “stay home”, “covid”, “corona”, “coronavirus”, “korona”, “covid-19”, “Covid19”, “Covid-19”, “Corona Virus”, “pandemic”, “pandemi”, “COVID-19”. Another parameter is “languages = “tr”,” which is used to extract only Turkish tweets. Received tweets were manually transferred to a table and manually marked there. In the second method used, when collecting tweets, they were drawn by searching for the word “pandemic” from random months from March 2020 to March 2021 with the snscrape library, and tweets collected at random time intervals were purified from those written as retweets, links, and replies.

The tweets created by the specified two methods were brought together. After collecting the tweets, they were subjected to various data preprocessing. First, collected tweets underwent the Noisy Data Cleaning process. The purpose of this process is to clear the data from unnecessary and nonsignificant data under sentiment analysis. Emojis links starting with HTTP, various symbols such as punctuation marks, and usernames starting with @ sign in tweets belonging to Twitter have been removed from tweets. After this process, the content of the tweet is completely converted to lowercase in order to edit the tweet. Afterward, the whitespaces at the beginning and end of the tweets were removed.

**Figure 3:**
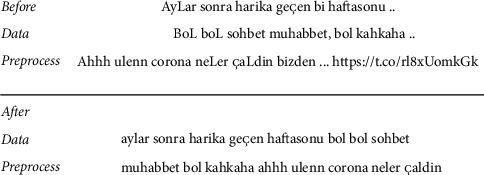
Data preprocessing example of cleaning noisy data and removing pause words.

The next data preprocessing is the deletion of stopwords. These words, which show language-specific variability, may vary in studies conducted in the literature. Within the scope of this study, stopwords are taken from the Turkish language stopword list created by Son [53] by combining the LUCENE-559 Turkish stopwords list and the “Information Retrieval on Turkish Texts” list. An example for preprocessing a tweet is shown in Figure 3.

**Figure 4:**
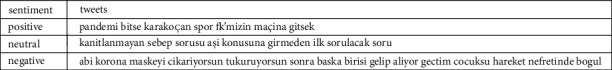
Sample data that belongs to the SentimentSet.

After combined tweets were preprocessed, they were marked manually, and SentimentSet was created. Tweets are marked as positive, negative, or neutral, which is shown in Figure 4. Tweets that express emotion but are not fully qualified and understood or that do not express positive or negative emotion are marked as neutral. Within the scope of the study, since the sentiment snalysis was based on positive or negative classification, tweets marked as neutral were not processed.

Before using these two datasets to train the models under the study, various data preprocessing processes are done to improve the data quality for the algorithms. At this stage, the preprocessing steps of Zeyrek [54] were used.

In this preprocess, firstly, noisy data cleaning and pause words are removed. These two operations do not affect the SentimentSet already prepared in this way. However, the dataset prepared by Beyaz [32] was thus passed through these processes.

**Figure 5:**
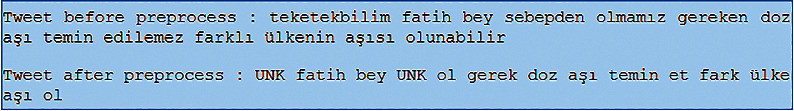
Example of word roots found using the Zemberek library.

Afterward, data stemming/lemmatization was performed. This process was performed using two different libraries. One of them is Zemberek [22] library, and the other is the SnowBall library belonging to NLTK. Zemberek [22] is a library written in java. With the help of the library's TurkishMorphology class, the lemmas of words were found with lemmatization. An example of word roots found with the help of Zemberek is shown in Figure 5.

**Figure 6:**
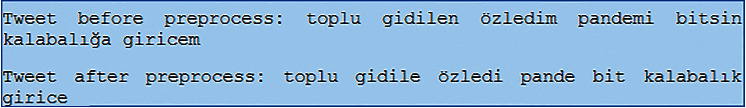
Example of word roots found using the snowball library.

The roots of the words were found by stemming using the class named TurkishStemmer belonging to the Snowball library of NLTK, another root-finding library used.  Figure 6 shows the rooting process by stemming with the Snowball library. With these libraries, root finding is carried out using two different methods, lemmatization and stemming. While creating the models, both methods were tried, and the results were reported.

After this process, another data preprocess, Text Vectorization, was applied to the data with roots. Before starting this process, the data must be separated as training and test data. In all models created within the scope of the study, 20% of the data was reserved for testing. The remainder was used for training. After the training and test data were separated, the Text Vectorization process was performed. The purpose of this process is simply to translate the data in human language, which underwent various preprocessing steps, into a language that the machine can understand. The data obtained as a result of this process are given as input to machine learning algorithms.

**Figure 7:**
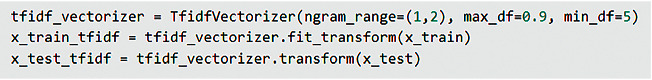
Tf-idf method representation used for Text Vectorization.

In this study, the TF-IDF technique was used for Text Vectorization.  Figure 7 shows how the Tf-idf technique is used. Since tf-idf is a bag of words technique, the “ngram_range = (1, 2)” parameter indicates that the unigram and bigram approaches of the word bag method are used together. The “max_df = 0.9” parameter shown in the figure means ignores terms that appear in more than 90% of the documents. Likewise, the “min_df = 5” parameter means ignore terms that appear in fewer than five documents. As shown in the figure, after the vectorizer object is created with the specified parameters, the fit_transform method is called to scale according to the “x_train” data reserved for training and to learn these scaling parameters. The mean and variance of the features of the training set are learned. Then, “x_test” is scaled according to these learned parameters by calling the transform() method. [55]

After the Text Vectorization process, the data was used in Machine Learning algorithms. However, these data preprocesses for LSTM are different from the others.

**Figure 8:**
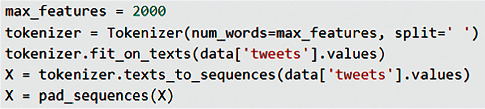
Tokenizer method notation used for LSTM.

In LSTM, Tokenization preprocessing is performed instead of rooting (stemming or lemmatization) and text vectorization. For this process, the Tokenizer class of the Keras library is used. [56] The Tokenizer class and parameters used for LSTM are shown in Figure 8. The value of “num_words = 2000” from the parameters shown in the figure determines how many words will be processed. Word separation operations were made according to the space with the given “ split = “ ” ” parameter. As seen in the figure, the dataset is given as a parameter to the fit_on_text method. Thus, the tokenizer has frequency information about the data. This method creates a word index based on frequency. Each word has its own integer value. The text_to_sequences in the figure replaces each word with the corresponding integer in the word_index dictionary. Pad_sequences in the figure is used for ensuring all sequences in a list have the same length. By default, this is done by adding 0 to the beginning of each sequence until each sequence has the same length as the longest sequence.

The data quality has been increased, and the data has been preprocessed and brought into a form that machine learning algorithms can use. Logistic Regression, SGD, Random Forest, Bayesian, SVM, and LSTM were used to train the models. The models created within the scope of the research are written in python language [57]. Google Colab was chosen as the working environment. The data prepared and preprocessed in the study carried out on Google Colab were used in many machine learning algorithms.

The Logistic Regression Model, which is one of the very common models in classification and regression problems, is used in the study. Model hyperparameters are used by default. The SGDClassifier model based on the Stochastic Gradient Descent algorithm is used in the study. This model's hyperparameters are used by default, except for the “max_iter = 5” parameter. The “max_iter” parameter, with a default value of 1000, indicates the maximum number of times to go over the data during training. The RandomForestClassifier model created with Random Forest Algorithm is used as one of the hyperparameters “n_estimators = 20”. This parameter, with a default value of 100, indicates how many trees are in the forest. If the other parameter used is “random_state = 0”, it is then added to ensure that the same result is obtained in every study. Other hyperparameters are used by default. The BernoulliNB model is used since the binary classification was made within the scope of the study. The model based on the Bayesian algorithm was used with the default hyperparameters.

The SVC model, which is a classification model of the Support Vector Machine algorithm, has been used as default with parameters other than “kernel = linear” one of the hyperparameters in the study. Since the data can be separated linearly, and the number of features extracted from the data is high, this parameter value is generally used in text classification problems [58]. The training dataset and the training vector were added to the models with the fit method. Prediction or classification was made on the test data by using the predict method on these models.

**Figure 9:**
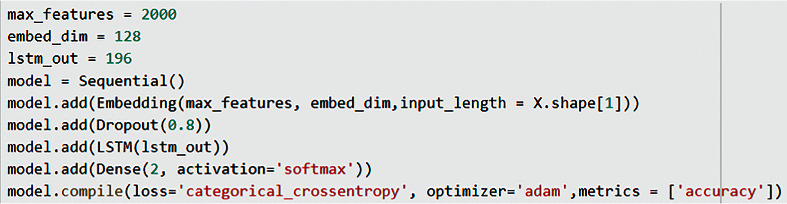
LSTM model and hyperparameters.

While creating the LSTM model, root finding and text vectorization were not applied in the data preprocessing step. After the noisy data cleaning and the removal of pause words, the previously mentioned tokenization data preprocessing method was used. The parameters of the Sequential model used are shown in Figure 9. An embedding layer, a dropout layer, an LSTM layer, and a Dense layer have been added to the model with the hyperparameters max_features, embed_dim, and lstm_out [59].

The Sequential model provides a sequential and layered structure. Each layer has an input and an output value. Layers are added to the model with the add method [60]

Embedding layer added that is frequently used for Keras text data to the model. Of the parameters in this method, “max_features” refers to the size of the vocabulary, and “input_length” refers to the length of the input strings. “embed_dim” defines the size of the vector space in which the words will be embedded. Furthermore, the “embed_dim” parameter expresses the size of the output vectors in the relevant layer for each word [61].

After the Dropout layer had been added to avoid the overfitting problem, the LSTM layer was added to the model. The lstm_out parameter used in this method represents the size of the output space. In the Dense layer, “2” is added as the first parameter because of the binary classification within the scope of the study. The second parameter is added as “sigmoid” that is used for the activation function.

**Figure 10:**
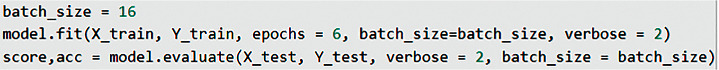
LSTM model fit and evaluate methods and parameters.

Finally, the “categorical_crossentropy” Adam algorithm and the accuracy metric have been added as parameters to the model compile method. Training data is added to this model with the fit method, as shown in Figure 10. The training data, the “epoch” number that shows the number of times to go over the whole dataset, the “batch_size” that represents the number of training samples used in each iteration, and the verbose parameter used to see a detailed output, have been added to the fit_method.

**Figure 11:**
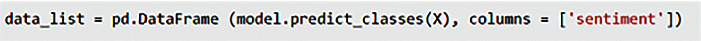
LSTM model predict_classes method.

Afterward, the test data is classified by using the evaluate method with the model. The method that gives the predicted classes by the model is shown in Figure 11. The evaluate method used in the model gives the loss function, while the predict method gives the predicted values.

## 4. Experimental Studies

**Figure 12:**
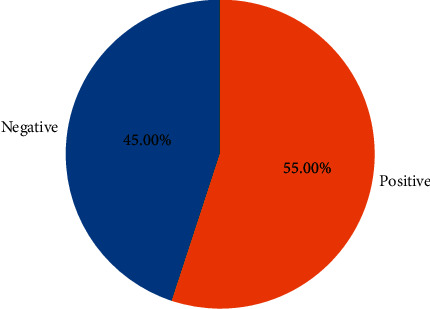
Visualization of the category ratios of the public dataset.

**Figure 13:**
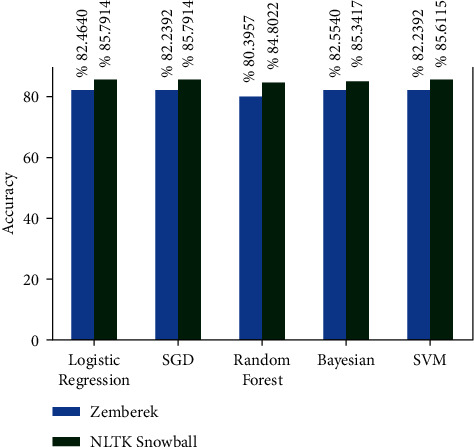
Comparison of accuracy rates of public dataset machine learning algorithms according to root-finding libraries.

There are approximately 11 thousand tweets in the public dataset [32]. The negative and positive category ratios of this dataset are shown in Figure 12. The results of the models created with the machine learning algorithms mentioned earlier on this dataset on the test data created by separating 20% of this dataset are shown in Figure 13. It shows the accuracy rates of these visual models and the comparison of results obtained when Zemberek or Snowball library is used as root-finding algorithms. It was observed that the success rates of the models are increased when the Snowball library belonging to NLTK was used as the root-finding algorithm with this dataset.

**Figure 14:**
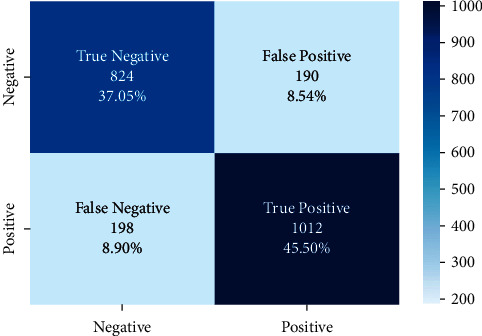
Distribution of test results and test data belonging to the dataset of Bayesian model trained with public dataset and Zemberek root-finding library.

**Figure 15:**
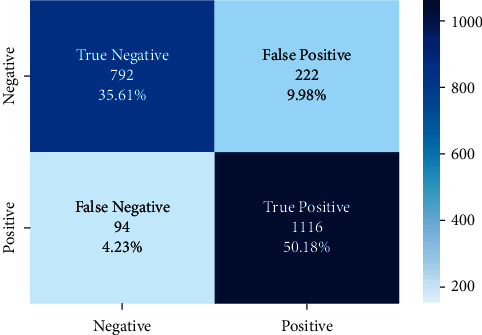
Distribution of test results and test data belonging to the dataset of logistic regression model trained with public dataset and Zemberek root-finding library.

The correct and incorrect predictions obtained when the Bayesian model is tested with the test data separated from the tilted models using the Zemberek library with the ready dataset are given in Figure 14. The correct and incorrect predictions are obtained when the Logistic Regression model, which is one of the models trained using the NLTK Snowball library with the ready dataset, is tested with the test data separated from the dataset. These are shown in Figure 15.

**Figure 16:**
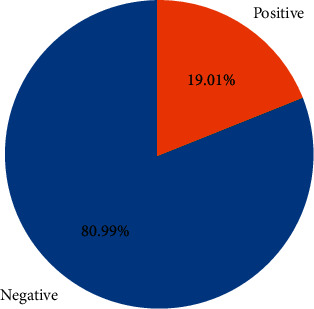
Visualization of the category ratios of the SentimentSet that is created in this study.

SentimentSet dataset, which has approximately 2600 tweets, is created within the scope of the study. When the neutral category was removed from this dataset, a dataset containing 2551 tweets with positive negative category ratios shown in Figure 16 was created. 20% of this dataset was reserved for test data, and 80% was used for training.

**Figure 17:**
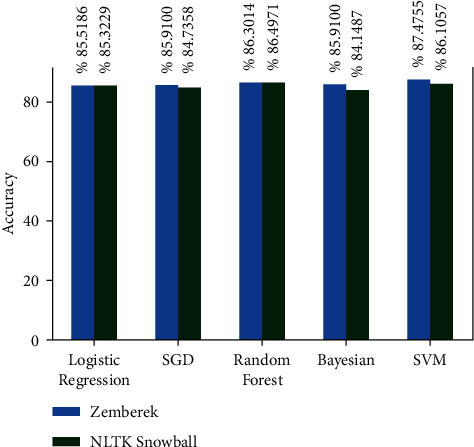
Comparison of the accuracy rates of SentimentSet and machine learning algorithms according to root-finding methods.

**Figure 18:**
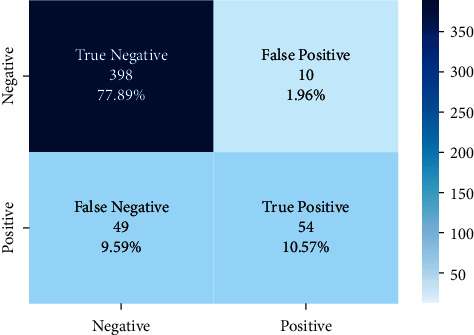
Distribution of test results and test data of SVM model trained with SentimentSet and Zemberek root-finding library.

**Figure 19:**
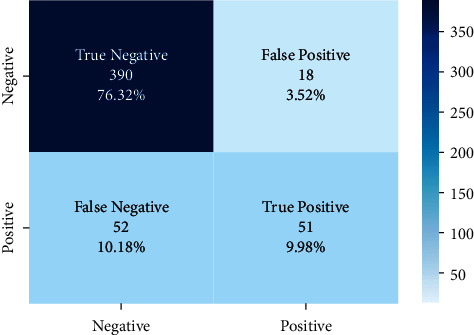
Distribution of test results and test data belonging to the dataset of random forest model trained with SentimentSet and NLTK snowball rooting library.

The results of the models created with the machine learning algorithms mentioned earlier on this dataset on the test data created by separating 20% of this dataset are shown in Figure 17. It shows the accuracy rates of these visual models and the comparison of results obtained when Zemberek or Snowball library is used as root-finding algorithms. It was observed that the success rate of the models increased when the Zemberek library was used as the root-finding algorithm with this dataset. The correct and incorrect predictions obtained when the SVM model is tested with the test data separated from the dataset, which is one of the best models using the Zemberek library with the SentimentSet, are given in Figure 18. The correct and incorrect predictions obtained when testing with the test data separated from the Random Forest model dataset, which is one of the models trained using the NLTK Snowball library with SentimentSet, are given in Figure 19.

**Figure 20:**
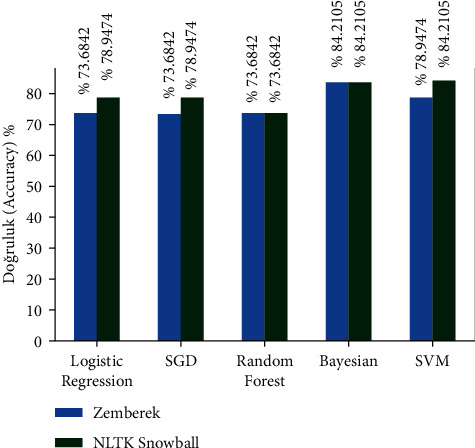
Test results with sample test data on models trained with SentimentSet.

**Figure 21:**
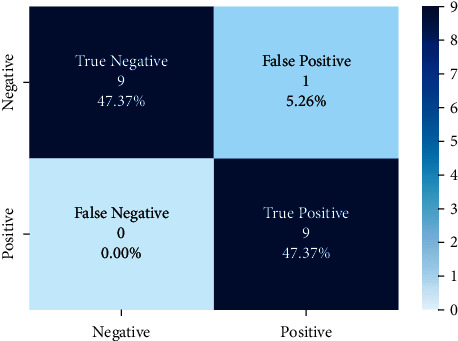
Distribution of test results with sample test data of Bayesian model trained with SentimentSet and Zemberek root-finding library.

**Figure 22:**
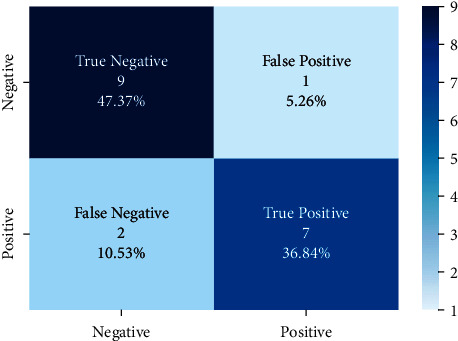
Distribution of test results with sample test data of SVM model trained with SentimentSet and NLTK snowball root-finding library.

Models trained with the SentimentSet were also tested with a small sample test data consisting of 20 randomly picked and marked nine positive and ten negative tweets that did not belong to the dataset but were generated by the same way of SentimentSet. The results obtained are shown in Figure 20 with the comparison of root-finding algorithms. The correct and incorrect predictions obtained when the Bayesian model is tested with sample test data, one of the best models using the Zemberek library with the SentimentSet, are given in Figure 21. The correct and incorrect predictions obtained when the SVM model, which is one of the models trained using the NLTK Snowball library with SentimentSet, is tested with the sample test data are given in Figure 22.

**Figure 23:**
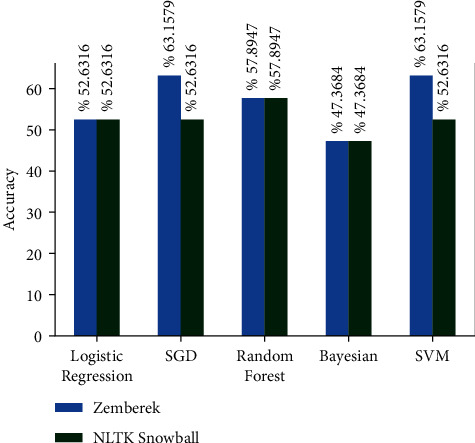
Test results with sample test data on models trained with the public dataset.

Since the aforementioned sample test data was created with the tweet collection method of SentimentSet described earlier within the scope of the study, the tests performed with this sample test data on the models trained with the ready dataset did not result in high accuracy rates, as shown in Figure 23.

**Figure 24:**
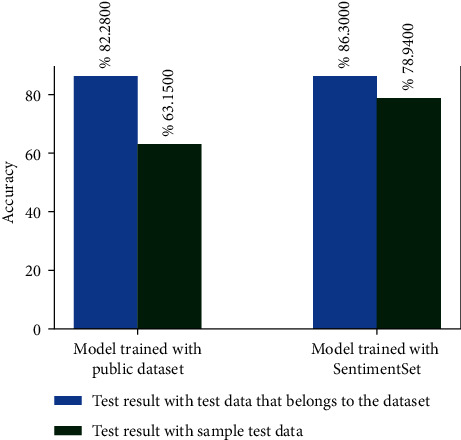
LSTM model training and test results with different datasets.

In the studies conducted with LSTM, root finding preprocess was not performed. While the model was trained, 20% of the dataset was reserved for the test data, and 80% of the dataset was used for training in the models created for both the public dataset and SentimentSet. The results obtained when the models trained using the public dataset or SentimentSet are tested with the test data of the dataset or with the sample test data consisting of 20 tweets are shown in Figure 24.

**Figure 25:**
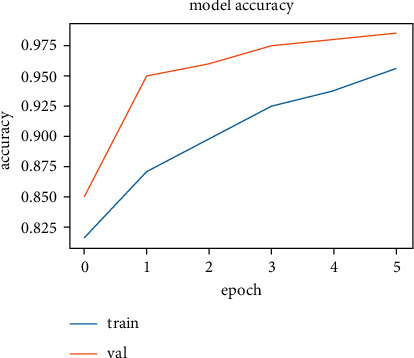
Training and validation accuracy values during LSTM model training with SentimentSet.

**Figure 26:**
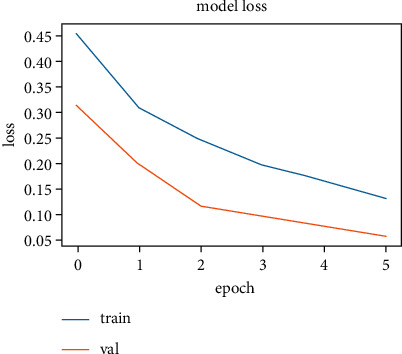
Training and validation error rate values during LSTM model training with SentimentSet.

The training and validation accuracy values obtained while training the LSTM model on the SentimentSet are shown in Figure 25, and the training and validation error rate values obtained during the training are shown in Figure 26.

**Figure 27:**
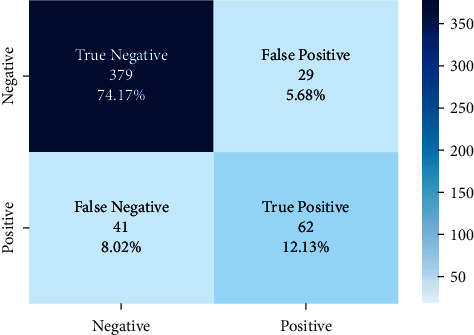
Distribution of test results and test data belonging to the dataset of the LSTM model trained with SentimentSet.

**Figure 28:**
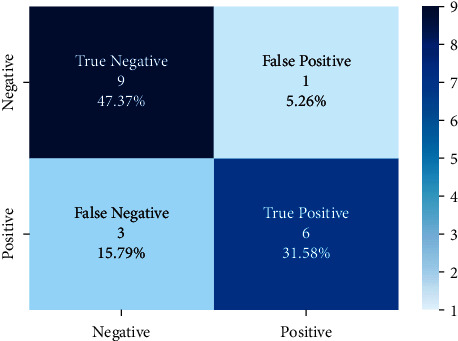
Distribution of test results with sample test data of the LSTM model trained with SentimentSet.

**Figure 29:**
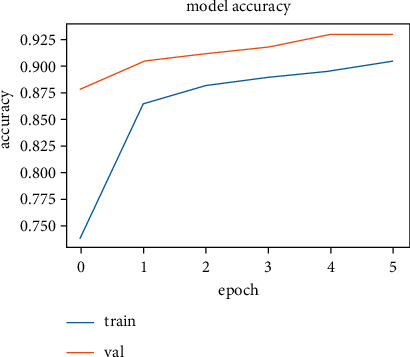
Training and validation accuracy values during LSTM model training with the public dataset.

**Figure 30:**
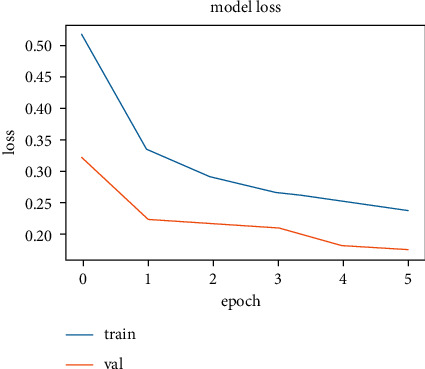
Training and validation error rate values during LSTM model training with the public dataset.

The correct and incorrect predictions obtained when the LSTM model trained with SentimentSet is tested with the test data that belongs to the dataset are given in Figure 27. The correct and incorrect predictions obtained when the LSTM model trained with SentimentSet is tested with the sample test data are given in Figure 28. Training and validation accuracy values during LSTM model training with the public dataset are shown in Figure 29. Training and validation error rate values during LSTM model training with the public dataset are shown in Figure 30.

**Figure 31:**
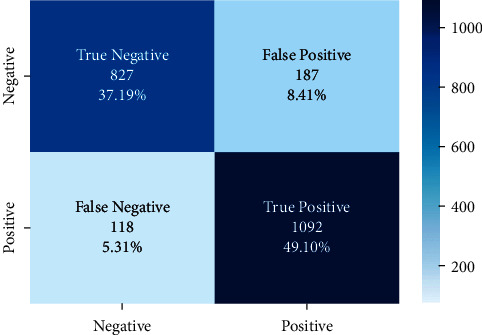
Distribution of test results and test data belonging to the dataset of the LSTM model trained with the public dataset.

**Figure 32:**
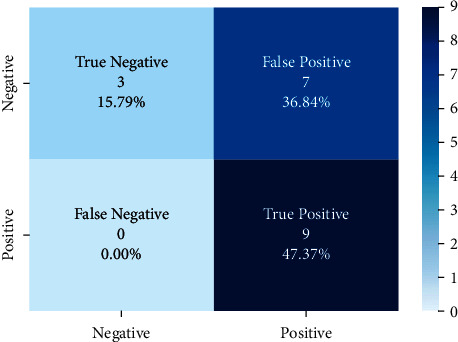
Distribution of test results with sample test data of the LSTM model trained with the public dataset.

The correct and incorrect predictions obtained when the LSTM model trained with the public dataset is tested with the test data that belongs to the dataset are given in Figure 31. The results obtained when the LSTM model trained with the public dataset is tested with the sample test data are shown in Figure 32.

In this study, various machine learning algorithms are used with different datasets and root-finding algorithms. All accuracy ratios of the combinations are shown in Table 2.

## 5. Discussion

In this study, we examined sentiment analysis for Turkish or different language in Twitter or in general in the literature over different topics. We observed that the dataset is one of the most challenging parts of sentiment analysis in Turkish. The public dataset [32] that we used is a general dataset that could be worked with different topics. Besides, we also aimed to contribute to adding benchmark dataset for “pandemic,” which is the hot topic recently. We made our study over these datasets, with using several machine learning algorithms. The results obtained by using different preprocessing techniques, different datasets for training and testing, and different machine learning algorithm combinations can be found in Table 2.

Furthermore, there are limited number of studies existing in the literature. Although there are not an enormous number of Turkish sentiment analysis studies, there are limited valuable studies in the literature. The accuracy results that belong to some of these studies from literature are given in Table 1.

Kirelli and Arslankaya [11] carried out a study that is Turkish sentiment analysis on global warming topic over 30000 random tweets from Twitter with using SVM, K-NN, and Bayesian. In that study, Hayran [27] used a dataset to train their models. The dataset was created with the labelling data based on emoticons; therefore, the accuracy could be little less than that of the manually labeled datasets. If we compare our study with some recent pandemic related Turkish language studies, we have nearly the same accuracy as the study [24] that used their own manually labeled benchmark dataset and worked with RNN, CNN, and HAN, which are deep learning models. However, our study differentiates with using different algorithms and datasets with different topics. On the other hand, another study [28] has very high accuracy over 15k tweets and CNN and bidirectional LSTM, which used a lexicon to label big number of data. As for us, in our study, we did not use lexicons; however, we used manually labeled dataset and SentimentSet that is created in the scope of the study. Our study contributes to the literature in many ways, but mainly it offers a general view of Turkish sentiment analysis accuracy on the several machine learning algorithms with SentimentSet, which could be used for future studies as benchmark dataset.

## 6. Conclusion

### 6.1. Theoretical Conclusion

With the development of technology day by day and the acceleration of artificial intelligence in the sector and academic studies, the diversity and number in this field in the literature are quite high today. Natural language processing, which is growing in use, is one of the topics of interest in this field. Social media are a very good data source for natural language processing studies because a wide variety of data can be accessed very quickly, and they are open source. Twitter API enables the anonymous use of Twitter users' tweets for academic studies or research after obtaining the necessary permissions. While the number of studies conducted in Turkish was very few in the past years, it is observed that it is increasing day by day.

Although Liu [62] says that it is also possible to make sentiment classification based on unsupervised learning, in this study, we preferred to make classification that is based on supervised learning with manually labeled datasets. Due to create SentimentSet, some words such as corona and pandemic were searched in the tweets. Likewise, when the sample data created with filters are tested with both models trained with the public dataset [32] and models trained with SentimentSet, it has been seen that the models created with SentimentSet are more successful. This shows that, in NLP tasks such as sentiment analysis, the similarity of the training data content and the test data content yields more successful results. The positive and negative category weights of the datasets used in the study are given. In the comparisons conducted on the same libraries and the same algorithms in the training phase carried with these datasets, it was seen that the negative prediction accuracy rate of SentimentSet with high negative data weight was higher than the positive prediction accuracy rate. It was observed that the positive prediction accuracy rate in the models trained with the ready dataset was higher than the SentimentSet. This confirms what is known that the dataset to have equal weights in classification problems is very important for machine learning algorithms to function in their best. However, it is more difficult to achieve these equal weights in real-life problems. Tweets with words such as “Pandemic” and “Corona” generally have a negative meaning. Hence, it is challenging to make manually labeled and equal weights in each class dataset with big amount of data on the other hand.

High negative data ratio of SentimentSet shows that there are some limitations choosing negative words like “pandemic” to collect tweets. While creating a custom dataset, it is ideal to have homogeneous labeled data with working on machine learning tasks. The more balanced dataset could give more accurate results in addition to SentimentSet dataset that was created for a periodic time that is approximately early of COVID-19 with limited data. Opinions about the words may vary because we are still in pandemic globally, and lots of changes have occurred since the beginning. Time period and data could be improved to get more valid and accurate results.

### 6.2. Practical Conclusion

Within the scope of the study, sentiment snalysis studies were carried out on Turkish tweets with natural language processing, and the results obtained by using various machine learning algorithms in this field were compared. Two different datasets, one public dataset [32] and the other one being SentimentSet [33] dataset that was created within the scope of the study with manually labelling, were used. These datasets were preprocessed before being used with algorithms. In the root-finding preprocess, which is one of these preprocesses, two different libraries were used and compared. In order to do sentiment analysis, these datasets were trained with Logistic Regression, SVM, Bayesian, Random Forest, and SGD algorithms, and models were produced. Apart from these algorithms, models were produced by training with datasets with LSTM, a deep learning network, and the results were compared in Table 1. This study is aimed to contribute to the natural language processing studies in the Turkish language in the literature. The trained models were tested with “Sample Test Data.” This test data was created separated from the datasets. “Sample Test Data” consists of 20 tweets collected with using the methods of creating the SentimentSet created within the scope of the study.

As seen in the study, the quality of the data is as important as the creation of models. On the other hand, the success of root-finding algorithms differs according to the dataset and the tested data. It is seen that the models trained with the SentimentSet have higher success rates with the test data separated from the dataset within itself compared to the models trained with the public dataset [32]. However, this may be a result of the negative category weight being too high in the SentimentSet. The models produced within the scope of the study can be improved, and models with better results can be produced. One of the ways to be followed for this is to increase the quality of the data. Solutions such as better filtering, detailing, and diversification of preprocesses can be produced.

Better training can be provided by changing the hyperparameters of the algorithms used in the study. The hyperparameters of the LSTM algorithm used in this study were determined with using trial and error method. In future studies, the most appropriate values can be determined by using methods such as genetic algorithms while selecting the hyperparameters of this model.

As a result, it is challenging to work with Turkish language because of the language specifications; for example, Turkish is an agglutinative language, so it requires different techniques from English to work with, and there are a limited number of studies in the literature. This study aimed to contribute as a Turkish language study to generating good overview to compare performances of machine learning algorithms with the different libraries and datasets. To make supervised learning, manually labeled datasets were used. One of them is SentimentSet dataset that could be used as benchmark dataset by future studies. Totally, with 2 datasets and 2 preprocessing techniques and different test data combinations, there are significant results that up to %87 are taken from the models. The results of the models are shown in Table 2.

The models and results created in this study show that machine learning algorithms in the Turkish language and sentiment analysis are promising and can be better in the future. It is, of course, possible to expand this success, to produce larger data and better models. Turkish studies can be developed by eliminating the weaknesses of the models and increasing the data quality. Thus, models that perform sentiment analysis tasks in Turkish can continue to influence our lives with much higher success rates and to develop with technology as it progresses.

## 7. Managerial Implication

The managerial implication of our research is that organizations can apply the proposed social analytics methodology to understand people's sentiment either pandemic or another topic that depends on people's opinion and hence improve their approach about their services to the people.

## 8. Practical/Social Implications

The findings could be used to understand how the pandemic affected people's sentiments from the tweets about this topic with given time duration. If this model is used for different timelines during the pandemic, it can be seen how people react with significant changes like COVID tests, vaccination, etc. Moreover, in general, with these public dataset [32] models, if they are used to make classification in another topic like world peace, women, or human rights, and if these results are shared with people, they could create solidarity about the event or topic and make their voices heard.

## 9. Limitations and Future Research

Although many machine learning algorithms including LSTM are used in this Turkish language study and taken satisfying results, better preprocessing and more balanced dataset are still needed to get better ones. In the results of algorithms, there is also a gap between accuracy of “sample test data,” which is independent from datasets that are used for training models, and “test data from datasets”; this can be seen in Table 2. This shows that there should be more qualified data to learn features more effectively.

Furthermore, while SentimentSet has limited amount of data, these custom datasets may contain more data in the future with applying lexicon-based approach to prevent manually labelling. In addition to data and preprocessing, studies [28, 46] that are conducted with LSTM and CNN have higher accuracy results; these techniques provide good results on sentiment analysis in English language and may also be used for Turkish language studies to get higher accuracy results.

As future work, new algorithm approaches including artificial neural networks with highly preprocessed balanced datasets could be done on Turkish social media sentiment analysis to maximize the accuracy.

## Figures and Tables

**Table 1:** Accuracy table of given studies that are conducted in the Turkish language.

Study	Accuracy
Kaya et al. [15]	%77
Akba [16]	%83.9 F1 score
Coban et al. [17]	%66.06
Karamollaoglu et al. [18]	%80
Bozyigit et al. [19]	%91 F1 score
Pervan [20]	%94.21
Rumelli et al. [21]	%73.8
Coltekin [23]	%77.3 F1 score
Kirelli and Arslankaya [11]	%74.63
Shehu et al. [24]	%88.8
Hayran and Sert [27]	%80.05
Kabakus [28]	%97.895

**Table 2:** Accuracy table of the algorithms with given combinations.

Accuracy table (%)	Zemberek				NLTK snowball			
	Train with public dataset		Train with SentimentSet		Train with public dataset		Train with SentimentSet	
	Test with public dataset	Test with sample test data	Test with SentimentSet	Test with sample test data	Test with public dataset	Test with sample test data	Test with SentimentSet	Test with sample test data
Logistic regression	%82.46	%52.63	%85.51	%73.68	%85.79	%52.63	%85.32	%78.94
SGD	%82.23	%63.15	%85.91	%73.68	%85.79	%52.63	%84.73	%78.94
Random forest	%80.39	%57.89	%86.30	%73.68	%84.80	%57.89	%86.49	%73.68
Bayesian	%82.55	%47.36	%85.91	%84.21	%85.34	%47.36	%84.14	%84.21
SVM	%82.23	%63.15	%87.47	%78.94	%85.61	%52.63	%86.10	%84.21

LSTM accuracy without using any root finding library (Zemberek or NLTK snowball)								
LSTM accuracy table (%)	Train with public dataset				Train with SentimentSet			
	Test with public dataset		Test with sample test data		Test with SentimentSet		Test with sample test data	
LSTM	%86.28		%63.15		%86.30		%78.94	

## Data Availability

The datasets used to support the findings of this study are available from the direct link in the dataset citations. The public dataset used within the scope of the study is an open dataset that can be accessed via https://www.kaggle.com/mrtbeyz/trke-sosyal-medya-paylam-veri-seti. The second dataset used is the SentimentSet, developed within the scope of this study, which can be accessed via https://www.kaggle.com/caglaballi/sentimentset.
